# Age-Dependent and -Independent Effects of Perivascular Adipose Tissue and Its Paracrine Activities during Neointima Formation

**DOI:** 10.3390/ijms21010282

**Published:** 2019-12-31

**Authors:** Eva Schütz, Rajinikanth Gogiraju, Maria Pavlaki, Ioannis Drosos, George S. Georgiadis, Christos Argyriou, Amina Rim Ben Hallou, Fotios Konstantinou, Dimitrios Mikroulis, Rebecca Schüler, Magdalena L. Bochenek, Sogol Gachkar, Katja Buschmann, Mareike Lankeit, Susanne H. Karbach, Thomas Münzel, Dimitrios Tziakas, Stavros Konstantinides, Katrin Schäfer

**Affiliations:** 1 Center for Cardiology, Cardiology I, University Medical Center Mainz, D-55131 Mainz, Germany; evamariaschuetz@web.de (E.S.); gogiraju@gmail.com (R.G.);; 2 Department of Cardiology, Democritus University of Thrace, GR-68100 Alexandroupolis, Greece or; 3 Department of Vascular Surgery, Democritus University of Thrace, GR-68100 Alexandroupolis, Greece; 4 Department of Cardiothoracic Surgery, Democritus University of Thrace, GR-68100 Alexandroupolis, Greece; 5 Center for Thrombosis and Hemostasis, University Medical Center Mainz, D-55131 Mainz, Germany; 6 Department of Cardiothoracic and Vascular Surgery, University Medical Center Mainz, D-55131 Mainz, Germany

**Keywords:** aging, atherosclerosis, neointima formation, perivascular adipose tissue

## Abstract

Cardiovascular risk factors may act by modulating the composition and function of the adventitia. Here we examine how age affects perivascular adipose tissue (PVAT) and its paracrine activities during neointima formation. Aortic tissue and PVAT or primary aortic smooth muscle cells from male C57BL/6JRj mice aged 52 weeks (“middle-aged”) were compared to tissue or cells from mice aged 16 weeks (“adult”). Vascular injury was induced at the carotid artery using 10% ferric chloride. Carotid arteries from the middle-aged mice exhibited smooth muscle de-differentiation and elevated senescence marker expression, and vascular injury further aggravated media and adventitia thickening. Perivascular transplantation of PVAT had no effect on these parameters, but age-independently reduced neointima formation and lumen stenosis. Quantitative PCR analysis revealed a blunted increase in senescence-associated proinflammatory changes in perivascular tissue compared to visceral adipose tissue and higher expression of mediators attenuating neointima formation. Elevated levels of protein inhibitor of activated STAT1 (PIAS1) and lower expression of STAT1- or NFκB-regulated genes involved in adipocyte differentiation, inflammation, and apoptosis/senescence were present in mouse PVAT, whereas PIAS1 was reduced in the PVAT of patients with atherosclerotic vessel disease. Our findings suggest that age affects adipose tissue and its paracrine vascular activities in a depot-specific manner. PIAS1 may mediate the age-independent vasculoprotective effects of perivascular fat.

## 1. Introduction

The importance of age as a cardiovascular risk factor is well established: epidemiological and clinical studies have shown that age is associated with increased cardiovascular mortality and risk of coronary heart disease, stroke, and death after myocardial infarction [1]. Age also increases the risk of developing restenosis, an important complication of intravascular revascularization procedures [2]. Elderly patients undergoing percutaneous coronary intervention not only exhibit more extensive and complex coronary arteriosclerosis [3], but large database analyses have revealed older age as one of the strongest predictors of acute complications and post-procedural outcome [4]. In addition, angiographic follow-up examinations have demonstrated significantly higher restenosis rates in elderly versus younger patients [5].

Regarding the underlying mechanisms, preclinical studies on the effects of age on the vascular response to injury have yielded inconsistent results: an increased neointima formation was reported in older rats [6,7] and mice [8], whereas injury was found to induce intimal thickening in young but not in older animals [9]. The mediators of the cardiovascular effects of age on intimal hyperplasia are also incompletely understood: age-associated endothelial dysfunction with subsequent failure to limit smooth muscle cell (SMC) proliferation, chronic low-grade inflammation, as well as elevated oxidative stress, has been suggested to increase the vulnerability of vascular tissues to stress [10]. Age-associated alterations of SMCs resulting in increased proliferative and migratory capacity [6] and an enhanced response to growth factor stimulation [8] may also play a role.

Experimental findings and clinical data suggest that vascular repair processes are modulated by the adventitia. The adventitia contains (myo)fibroblasts and SMC precursors that migrate towards the media and neointima following stimulation [11], and immune cells are recruited to the vessel wall via perivascular blood vessels [12]. Immediately adjacent to the adventitia of all larger arteries lies the perivascular adipose tissue (PVAT). Previous studies, including those by our group, have shown that cardiovascular risk factors alter the phenotype, composition, and function of PVAT, resulting in loss of its protective properties [13,14]. However, little is known about the impact of age on perivascular fat and whether and how it affects the response to vascular injury.

In this study, we examine age-associated alterations of different adipose tissue depots including perivascular fat and whether they contribute to vascular remodeling processes. We employ models of neointima formation in adult and middle-aged mice, murine preadipocyte differentiation in vitro and also investigate perivascular adipose tissue specimens from older patients with atherosclerotic vessel disease undergoing surgery.

## 2. Results

### 2.1. Middle-Aged Mice Exhibit Visceral Adiposity, Prediabetes, and Perivascular Adipocyte Hypertrophy

To examine the effect of age on the vascular wall including perivascular adipose tissue, male C57BL/6JRj WT mice aged 52 weeks (hereafter termed “middle-aged”; *n* = 20 mice) were compared to male C57BL/6JRj WT mice aged 16 weeks (“adult”; *n* = 20 mice). Aging was associated with increased mean body weight and visceral adiposity (Table 1). PVAT could not be weighed, but morphometric analysis revealed a significantly increased mean single adipocyte area in middle-aged mice (Supplemental Figure S1A), and similar findings were observed in VAT (Supplemental Figure S1B) and BAT (Supplemental Figure S1C). Representative H&E-stained cross-sections are shown in Supplemental Figure S1D. Analysis of metabolic serum parameters after overnight fast (*n* = 15 mice per group) revealed significantly elevated glucose levels in middle-aged mice (Table 1). Serum leptin, insulin levels and the HOMA-IR index, or total, HDL and LDL cholesterol levels did not differ (Table 1).

### 2.2. Age is Associated with Smooth Muscle Cell Senescence and De-Differentiation

**Figure 1:**
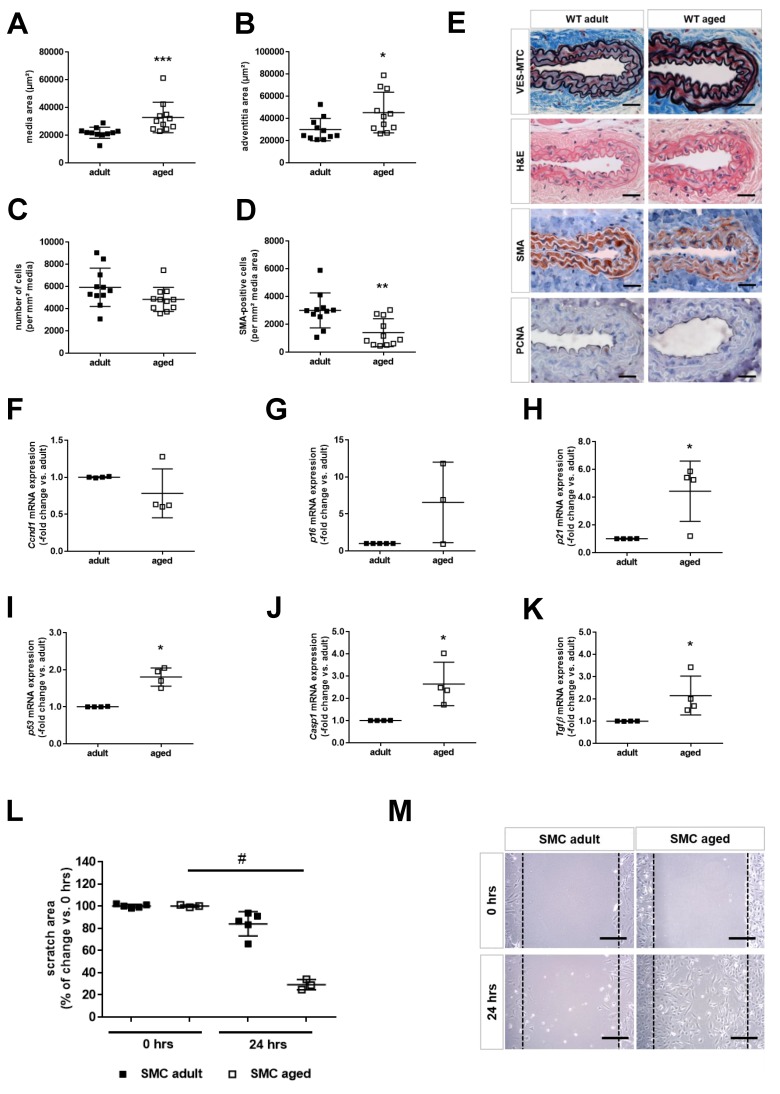
Age-associated changes of the arterial vessel wall. Cross-sections through the uninjured common carotid artery of adult (*n* = 11) and middle-aged (*n* = 11) mice were stained with VES-MTC to visualize elastic fibers (black), muscle cells (red) and extracellular matrix (blue), or with H&E to visualize cell nuclei (blue-black). Antibodies against smooth muscle alpha-actin (SMA) or proliferating cell nuclear antigen (PCNA) were used to immunostain smooth muscle cells (red) or proliferating cells (brown), respectively. The media area (**A**) and adventitia area (**B**) were quantified using ImagePro Plus analysis software. The total number of cells was counted manually and expressed per mm² media area (**C**). The SMA-immunopositive area per total media area was determined using the count-size function (**D**). Individual data points and the mean ± SD are shown. * *p* < 0.05, ** *p* < 0.01 and *** *p* < 0.001 vs. adult mice (as determined by Student’s *t*-test for unpaired means (in B) or the Mann–Whitney test (in **A**,**C**,**D**)). Representative findings are shown in (**E**). Size bars represent 100 µm. Total mRNA was isolated from the aorta of adult (*n* = 4) and middle-aged (*n* = 4) mice and the mRNA expression of cyclin D1 (Ccnd1; **F**), p16INK4a (p16; **G**), p21Cip1 (**H**), p53 (**I**), caspase-1 (Casp1; **J**) and transforming growth factor-beta (Tgfβ; **K**) was determined using real-time PCR. * *p* < 0.05 vs. adult mice (as determined by the Mann–Whitney test). Primary smooth muscle cells (SMCs) were isolated from the aorta of adult (*n* = 5) and middle-aged (*n* = 3) mice and their proliferative and migratory activities analyzed using the scratch-wound assay. The summary of the quantitative is shown in (**L**), representative findings at 0 and 24 h after scratch injury in (**M**). Individual data points and the mean ± SD are shown. # *p* < 0.05 vs. adult mice (as determined by the Kruskall–Wallis test followed by Dunn’s multiple comparisons test). Size bars in panel M represent 100 µm.

VES-MTC staining of cross-sections through the uninjured carotid artery of adult and middle-aged mice (*n* = 11 mice per group) revealed that aging was associated with an increased media (Figure 1A), total vessel (*p* < 0.05) and adventitia (Figure 1B) area. H&E staining showed a reduced cell density (*p* = 0.056; Figure 1C) and a non-significant trend towards lower numbers of PCNA-positive, proliferating cells (6.2 ± 3.5% vs. 19.8 ± 10.9% of total cells, *p* = 0.176) in the media of middle-aged compared to adult mice, and the number of SMA-positive, differentiated SMCs was significantly reduced (Figure 1D). Representative images after VES-MTC, H&E, SMA, or PCNA staining are shown in Figure 1E. Quantitative PCR analysis of mRNA isolated from the thoracic aorta (*n* = 4 mice per group) revealed that messenger RNA levels of cyclin D1 (Figure 1F) in the arterial wall of middle-aged mice did not differ from those in adult mice, whereas mRNA levels of the senescence markers p16INK4A (Figure 1G), p21Cip1 (Figure 1H), and p53 (Figure 1I) were found to be increased in aortas of middle-aged compared to adult mice. Caspase-1, a marker of inflammasome activation (Figure 1J), and transforming growth factor-beta (TGFβ; Figure 1K) also were expressed at significantly higher levels. Of note, histochemical detection of senescence-associated β-galactosidase (SA-β-Gal) activity did not reveal positive cells in the uninjured carotid artery vessel wall of middle-aged mice, and Sudan black B staining also showed only a minimal increase in the presence of lipofuscin-containing cells (Supplemental Figure S2). Analysis of primary SMCs revealed a significantly accelerated wound closure 24 h after scratch injury in those isolated from the aorta of middle-aged mice (*n* = 3) compared to those from adult mice (*n* = 5). The summary of the quantitative analysis is shown in Figure 1L; representative findings in Figure 1M.

### 2.3. Aging Aggravates Media Thickening and Outward Remodeling after Arterial Injury

**Figure 2:**
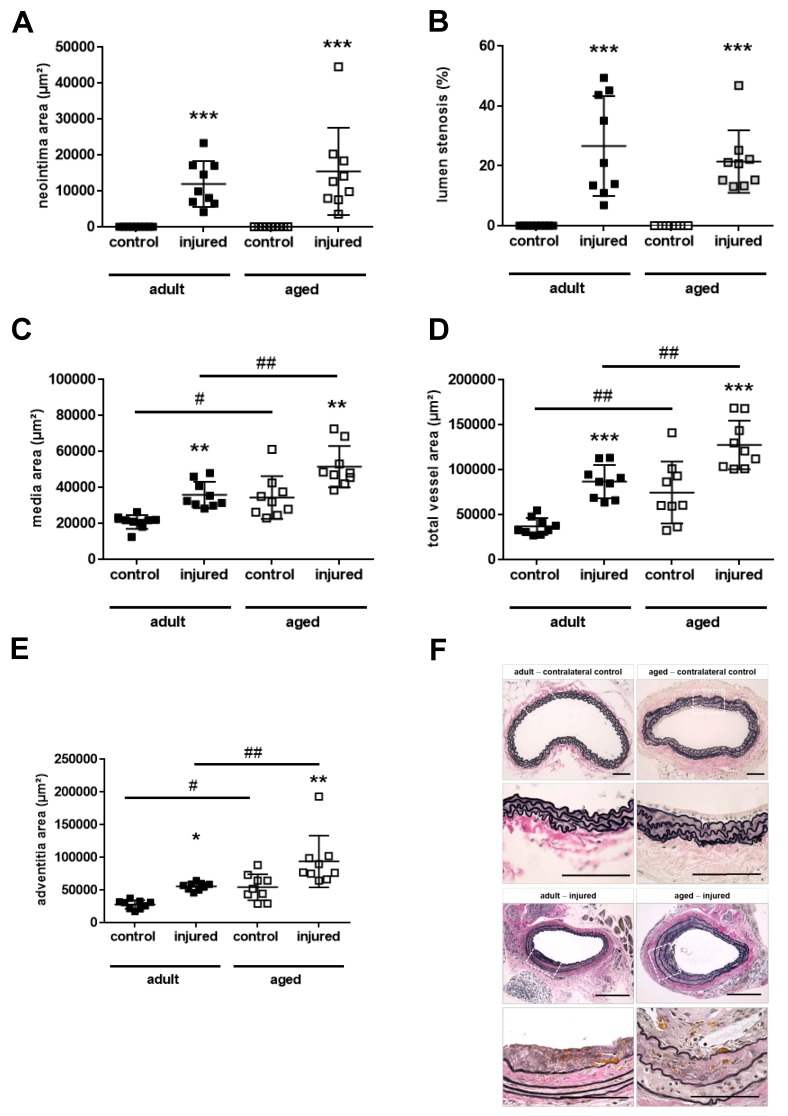
Neointima formation in adult and middle-aged mice. Vascular injury was induced at the carotid artery of C57BL6/JRj WT mice aged 16 weeks (adult; *n* = 9) or 52 weeks (middle-aged; *n* = 9). The neointima area (**A**), lumen stenosis (**B**), media area (**C**), total vessel area (**D**) and adventitia area (**E**) were quantified on at least three Verhoeff’s elastica (VES)-stained paraffin cross-sections through the injured carotid artery as well as the contralateral, uninjured artery (control), and results averaged per mouse. Individual mean values per mouse and the mean ± SD are shown. * *p* < 0.05, ** *p* < 0.01 and *** *p* < 0.001 vs. controls and # *p* < 0.05 and ## *p* < 0.01 vs. adult mice (as determined by the Kruskall–Wallis, followed by Dunn’s multiple comparisons test (**A**,**B**,**E**) or one-way ANOVA, followed by Sidak’s multiple comparisons test (**C**,**D**). Representative images in adult and middle-aged mice are shown in (**F**). Size bars represent 100 µm.

Previous studies in mice and rats have examined the effects of age on neointima formation, with inconsistent results [6,7,8,9]. To determine how age and the age-associated changes of the vessel wall affect vascular remodeling processes in our model, adult and middle-aged mice were subjected to carotid artery injury using 10% ferric chloride (*n* = 9 mice per group). Age had no effect on the neointima area (Figure 2A) or the degree of lumen stenosis (Figure 2B), but further enhanced the above-mentioned existing differences in the media (Figure 2C), total vessel (Figure 2D), and adventitia (Figure 2E) area. Representative images of VES-stained cross-sections through uninjured, contralateral control carotid arteries and vascular lesions of adult and middle-aged mice are shown in Figure 2F.

### 2.4. Transplantation of Perivascular Adipose Tissue Does Not Mimic the Vascular Aging Phenotype, but Age-Independently Reduces Neointima Formation and Lumen Stenosis

**Figure 3:**
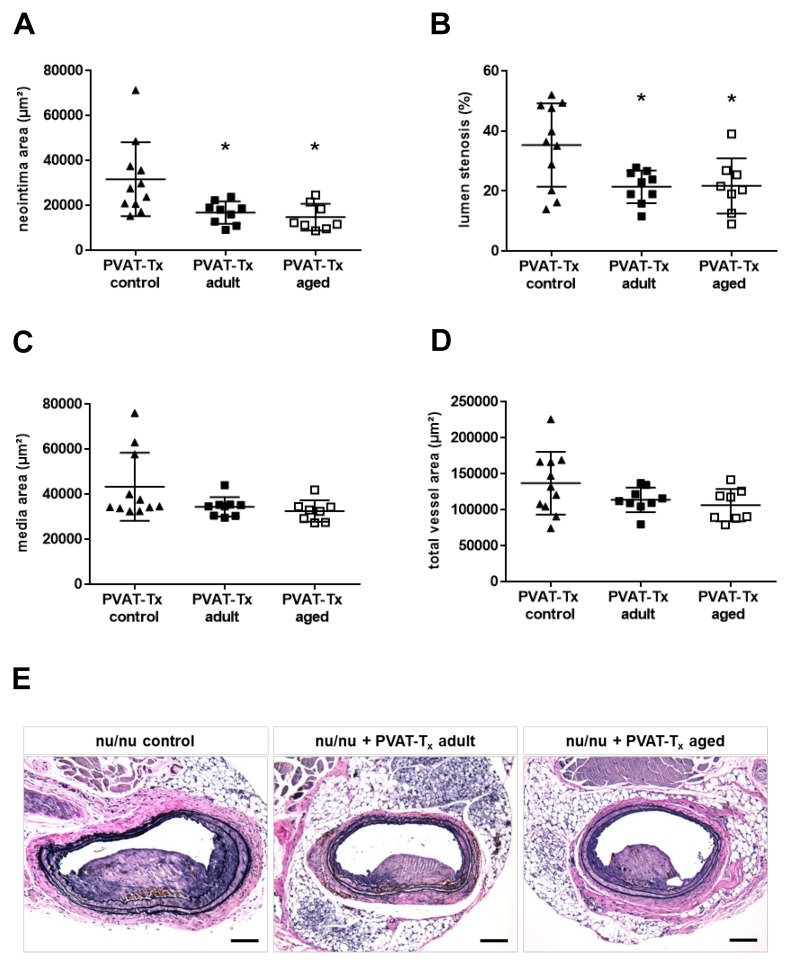
Effect of perivascular adipose tissue age on neointima formation. Vascular injury was induced at the carotid artery of NMRI nu/nu mice aged ten weeks immediately followed by transplantation (Tx) of PVAT freshly harvested from the thoracic aorta of adult (*n* = 9) or middle-aged (*n* = 8) C57BL6JRj mice. Nu/nu mice with their own PVAT left intact and not transplanted were used as control. Three weeks later, the neointima area (**A**), lumen stenosis (**B**), media area (**C**) and total vessel area (**D**) was morphometrically quantified on at least three VES-stained cross-sections and results averaged per mouse. Individual mean values per mouse and the mean ± SD are shown. * *p* < 0.05 vs. NMRI nu/nu not transplanted with PVAT (control; *n* = 11), as determined by the Kruskall-Wallis (**A**,**C**) or one-way ANOVA (**B**,**D**) test. Representative images are shown in (**E**). Size bars represent 100 µm.

Previous studies employing visceral fat transplantation suggested that adipose tissue surrounding the arterial wall participates in the vascular remodeling response to injury [15]. To determine the importance of age-associated changes of the adventitia and PVAT for neointima formation, an injury was induced in 12-week-old immune-incompetent NMRI nu/nu mice, immediately followed by transplantation of PVAT freshly removed from the thoracic aorta of adult (*n* = 9) or middle-aged (*n* = 8) donor mice to the site of vascular injury. PVAT surrounding the thoracic aorta was chosen as a substitute for carotid artery perivascular fat (which was not available in sufficient amounts) because of its anatomical proximity to the carotid artery, the similarity of their embryonic origin, and in order to avoid contamination with visceral fat. The mean age and body weight of the NMRI nu/nu host and C57BL/6J donor mice are given in Supplemental Table S1. Perivascular transplantation of PVAT after vascular injury did not significantly alter the media (Figure 3C) and total vessel (Figure 3D) area, i.e., the parameters found to be affected by age, compared to controls (*n* = 11 NMRI nu/nu mice not transplanted with PVAT). On the other hand, it significantly reduced the size of the neointima (Figure 3A) and the degree of lumen stenosis (Figure 3B), independent of the donor age. Representative findings of carotid arteries, including the transplanted aortic perivascular fat three weeks after injury, are shown in Figure 3E. These in vivo findings suggest that PVAT may exert age-independent protective effects, reducing neointima thickening and luminal stenosis.

### 2.5. Differential Expression of Potential Mediators of Neointima Formation in Perivascular and Visceral Adipose Tissue

**Figure 4:**
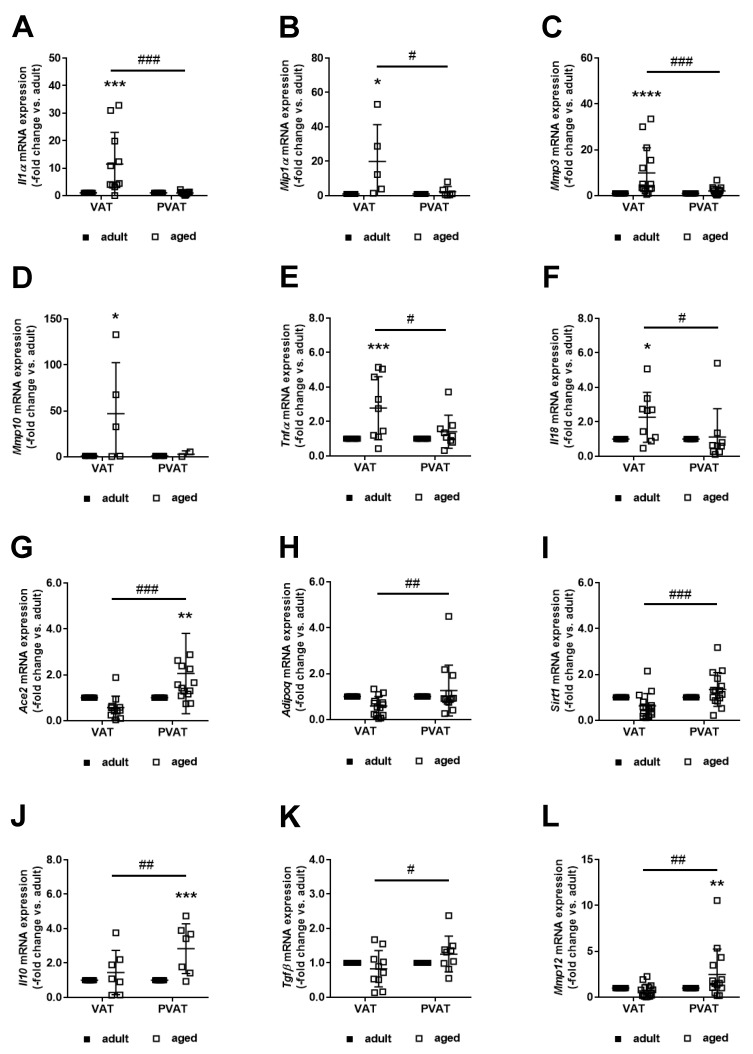
Differential effects of age on adipose tissue gene expression patterns. Visceral (VAT) and perivascular (PVAT) adipose tissues were harvested from adult (*n* = 5–16) and aged (*n* = 5–16) mice and total RNA isolated. Real-time PCR analysis was employed to quantify the mRNA expression of (**A**) interleukin-1alpha (Il1α), (**B**) macrophage inflammatory protein 1-alpha (Mip1α), (**C**) matrix metalloproteinase-3 (Mmp3), (**D**) Mmp10, (**E**) tumor necrosis factor-alpha (Tnfα), (**F**) Il18, (**G**) angiotensin-converting enzyme-2 (Ace2), (**H**) adiponectin (Adipoq), (**I**) sirtuin-1 (Sirt1), (**J**) Il10, (**K**) transforming growth factor-beta (Tgfβ), and (**L**) Mmp12. Individual data points and the mean ± SD are shown. * *p* < 0.05, ** *p* < 0.01, *** *p* < 0.001 and **** *p* < 0.0001 vs. adult mice and # *p* < 0.05, ## *p* < 0.01 and ### *p* < 0.001 vs. VAT (as determined by two-way ANOVA).

To identify potential candidate(s) mediating the protective effects of thoracic aorta PVAT during neointima formation, factors shown to affect vascular remodeling processes in a paracrine manner, or to be modulated by age, were examined using quantitative real-time PCR. Because VAT had been used for perivascular transplantation experiments in previous studies [14,16], findings in PVAT of adult and middle-aged mice were compared to those in their VAT. Messenger RNA levels of the senescence-associated secretory phenotype (SASP) markers IL1α (Figure 4A), MIP1α (Figure 4B), MMP3 (Figure 4C), MMP10 (Figure 4D), and TNFα (Figure 4E) were significantly increased in VAT of middle-aged compared to adult mice, whereas their expression was unchanged in their PVAT. Absence of an age-associated increase was also observed for IL18 (Figure 4F), an inflammatory cytokine shown to promote restenosis [17]. On the other hand, mRNA levels of factors previously shown to reduce neointima formation, such as ACE2 [18] (Figure 4G), adiponectin [19] (Figure 4H), SIRT1 [20] (Figure 4I), IL10 [21] (Figure 4J), or TGFβ [22] (Figure 4K), increased significantly with age only in PVAT. Although mRNA levels of MCP1, produced in (perivascular) adipose tissue and shown to promote neointima formation in a paracrine manner [23], did not differ (not shown), significantly increased mRNA levels of MMP12, involved in the generation of antagonists of MCP1 and other chemokines [24], were observed in PVAT of middle-aged mice compared to adult mice (Figure 4L). The absence of a SASP response in PVAT of middle-aged mice and higher expression of protective factors may explain why its transplantation reduced neointima formation and lumen stenosis in the underlying vessel.

### 2.6. Increased Expression of PIAS1 May Mediate the Anti-Inflammatory Expression Profile of Perivascular Fat

**Figure 5:**
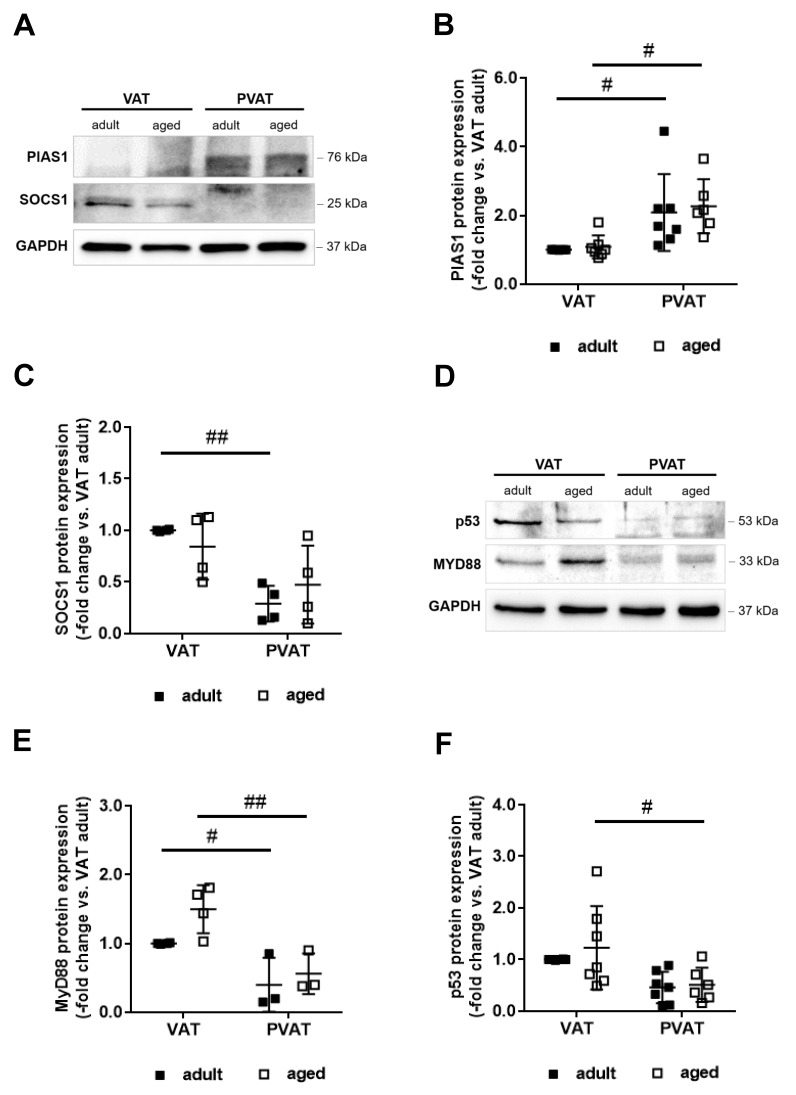
Perivascular adipose tissue expression of negative regulators of STAT1 and NFκB. (**A**,**B**) Western blot analysis of protein inhibitor of activated STAT-1 (PIAS1), (**A**,**C**) suppressor of cytokine signaling-1 (SOCS1), (**D**,**E**) myeloid differentiation primary response-88 (MyD88), and (**D**,**F**) tumor suppressor p53 (p53) in visceral (VAT) and perivascular (PVAT) adipose tissue of adult (*n* = 3–8) and middle-aged (*n* = 3–8) mice. Results were normalized to GAPDH protein levels and are expressed as -fold change vs. findings obtained in VAT of adult mice. Individual data points and the mean ± SD are shown. # *p* < 0.05 and ## *p* < 0.01 vs. VAT (as determined using two-way ANOVA). Representative Western blot membranes are shown in panels (**A**,**D**).

Several of the mediators found to be differentially expressed in PVAT and VAT are regulated by NFκB and/or STAT transcription factors. A common regulator and potent suppressor of both STAT1 transcriptional activity [25] and NFκB [26] signaling expressed in adipocytes is the protein inhibitor of activated STAT1 (PIAS1). To begin to examine the possibility that differences in PIAS1 may underlie the blunted inflammatory marker expression observed in PVAT, a Western blot analysis was performed, revealing significantly elevated protein levels of PIAS1 in PVAT compared to VAT (Figure 5A,B). A lower protein expression of SOCS1 indicated reduced STAT1 activation (Figure 5A,C), and reduced levels of MyD88 reduced NFκB signaling (Figure 5D,E). Expression of the tumor suppressor and senescence mediator p53 also was lower in PVAT (Figure 5D,F), in line with the PIAS1 function as an E3 SUMO ligase repressing p53 activity [27]. SiRNA-mediated downregulation of PIAS1 in murine 3T3L1 adipocytes increased the expression of SOCS1, MyD88, and p53 protein (Supplemental Figure S3A–D), supporting a causal role of PIAS1 in the differential expression of NFκB- and STAT-regulated genes in PVAT and VAT of mice.

### 2.7. Differences in Adipocyte Differentiation May Underlie Higher PIAS1 Expression in PVAT

STAT1 is involved, among others, in the transcriptional control of adipocyte differentiation [28]. To begin to study the mechanisms underlying the differential expression of PIAS1 in mouse VAT and PVAT, murine 3T3L1 preadipocytes were examined at baseline and at different time points after induction of adipogenesis. Adipogenic differentiation was verified morphologically and by Oil red O staining (Supplemental Figure S4A,B) and increased expression of perilipin (Supplemental Figure S4C,D). PIAS1 protein expression was highest in 3T3L1 preadipocytes and gradually decreased with adipogenic differentiation (Supplemental Figure S5A,B). In parallel with the reduced expression of PIAS1, increased mRNA levels of STAT1 (Supplemental Figure S6A) and STAT1-regulated genes involved in adipocyte differentiation, such as leptin (Lep; Supplemental Figure S6B), sirtuin-1 (Sirt1; Supplemental Figure S6C) or lipocalin-2 (Lcn2; Supplemental Figure S6D), were observed. In line with our observation of increased PIAS1 levels in murine PVAT in vivo and preadipocytes in vitro, the preadipocyte marker PREF1 was expressed at higher levels in PVAT of adult and middle-aged mice compared to their VAT, whereas protein levels of the mature adipocyte marker perilipin-1 and the adipokine leptin (were lower in PVAT compared to VAT (representative Western blot membrane shown in Supplemental Figure S7A). Findings were confirmed on the gene expression level showing significantly increased Pref1 (Supplemental Figure S7B) and reduced leptin (Supplemental Figure S7C) mRNA levels in PVAT compared to VAT.

### 2.8. Reduced PIAS Levels in Human Perivascular Adipose Tissue Surrounding Atherosclerotic Vessels

**Figure 6:**
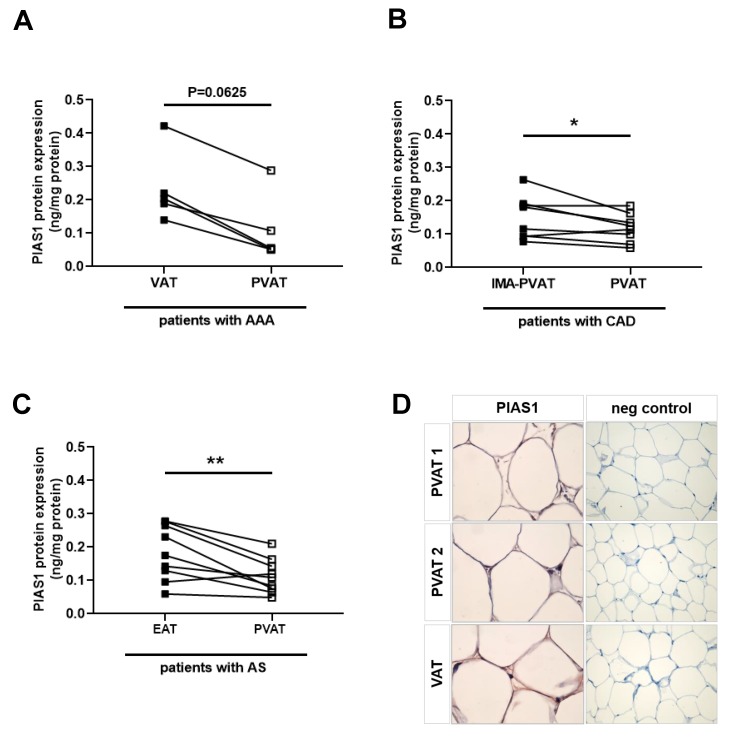
Expression of PIAS1 in human perivascular adipose tissue. Quantitative analysis of PIAS1 protein levels in visceral (VAT) and perivascular (PVAT) adipose tissue surrounding the aorta of patients (*n* = 5) with abdominal aortic aneurysm (AAA) using an enzyme-linked immunoassay (**A**). Results are expressed per ng/mg total protein. Quantitative analysis of PIAS1 in PVAT surrounding the aortic root and coronary arteries of patients (*n* = 8) with coronary artery disease (CAD) compared to PVAT surrounding the internal mammary artery (IMA-PVAT) (**B**). Quantitative analysis of PIAS1 in PVAT surrounding the aortic root and coronary arteries of patients (*n* = 9) with aortic valve stenosis (AS) compared to epicardial adipose tissue (EAT) from the same patient (**C**). Exact *p*-values, as determined using the Wilcoxon matched-pairs signed rank test (**A**) or paired Student’s *t*-test (**B**,**C**) are shown within the graphs. Immunohistochemical detection of PIAS1 (red signal) in human VAT and PVAT (×400 magnification) (**D**). Results after the omission of the first antibody (neg control) are also shown (×200 magnification).

Loss of protective properties and proinflammatory changes in PVAT may contribute to the progression of vascular disease [15]. To begin to translate our observations in mice to human pathologies, PVAT and VAT were obtained from patients with atherosclerotic vessel disease (a manifestation of vascular aging) and examined for the expression of PIAS1. Patients with abdominal aortic aneurysm (AAA) were chosen for these analyses because they provide the opportunity to obtain both visceral and periaortic adipose tissue during a single surgical intervention (laparotomy) and to compare them within the same individual. Opposite to our findings in atheroprotective PVAT of mice, reduced PIAS1 protein levels (as determined by ELISA to determine absolute protein amounts) were observed in PVAT surrounding the diseased human abdominal aorta compared to their VAT (Figure 6A). Significantly reduced PIAS1 levels were also observed in PVAT surrounding the aortic root and coronary arteries of patients with coronary artery disease compared to PVAT surrounding the internal mammary artery (IMA), a vessel largely protected from arteriosclerosis [29] (Figure 6B). Also in PVAT of patients with aortic stenosis, PIAS1 protein levels were found to be expressed at significantly lower levels compared to their epicardial adipose tissue (EAT; Figure 6C), and the PIAS1 immunosignal could be localized, among other cells, to adipocytes (Figure 6D). Although these observations in humans with the atherosclerotic disease cannot prove causality, they support our findings in mice and suggest that reduced expression of PIAS1 may participate in age-associated vascular disease processes.

## 3. Discussion

Studies in humans and animal models have examined the effects of age on vessel walls and found it to be associated with distinct phenotypical changes. We now sought to extend those previous findings by examining the impact of age on perivascular adipose tissue and its paracrine activities during the vascular remodeling response to injury. Although we observed an age-associated enlargement of the adventitia and perivascular adipocyte hypertrophy, transplantation of PVAT around the injured murine carotid artery was found to reduce neointima formation independent of the mouse donor age. In this regard, molecular analyses suggested specific “protective” alterations in murine PVAT with age, which were distinct from changes in visceral fat and may have contributed to the observed reduced neointima formation following its transplantation around the injured murine carotid artery. Specifically, potentially proatherogenic changes present in VAT, such as increased expression of TNFα, IL1α, MIP1α, MMP3, or MMP10 with age, were absent in thoracic PVAT, whereas the expression of potential mediators involved in atheroprotection, such as adiponectin, MMP12, or IL10, were expressed at significantly higher levels in aged PVAT compared to aged VAT. Independent of age, higher expression of PIAS1 (a key negative regulator of inflammation) but also adipocyte differentiation was observed in PVAT compared to VAT, whereas PIAS1 protein levels were reduced in PVAT of patients with atherosclerotic aortic aneurysms, coronary artery disease, or aortic stenosis. These findings suggest differential, depot-specific effects of age on adipose tissue and its paracrine activities on the vessel wall, and also support a critical role of PIAS1 in controlling perivascular adipose tissue differentiation and inflammatory and atherogenic marker expression.

Age-associated alterations of the vasculature may render the cardiovascular system susceptible to disease, even in the absence of additional risk factors. In this study, middle-aged mice exhibited a significantly larger carotid media and total vessel area compared to younger mice, and these differences became even more pronounced following vascular injury. Previous studies using rats or mice 18 months or older have examined the effect of age on the response to arterial injury, however, with inconsistent results [6,8,9]. Interestingly, transplantation of aortic grafts from old rats into young recipients resulted in greater neointima formation [6]. Others found young aortas develop thicker neointimas when transplanted into old recipients, whereas the opposite was observed after transplantation of old aortas in young recipients [30]. Our finding that transplantation of PVAT did not mimic the effects of age on neointima formation may be interpreted as pointing to a predominant role of the vessel wall itself, or systemic factors, in response to injury with age.

Neointima formation following vascular injury is the result of the migration and proliferation of vascular SMCs from the media and the adventitia, and aging was found to accelerate and prolong the proliferative capacity of SMCs [31]. Increased TGFβ activation [32] or PDGF receptor expression and a stronger response to PDGF stimulation compared to young SMCs [8] have been identified as potential mechanisms. Others found either no increase in the growth capacity of vascular SMCs with age [33] or reduced proliferation of SMCs isolated from arteries of old rats and increased apoptotic cell death after balloon injury [34]. In our study, markers of cell proliferation, such as PCNA or Ccnd1, did not differ, whereas factors associated with senescence, such as p21Cip1 or p53, were expressed at significantly higher levels in the aorta of middle-aged compared to adult mice. Of note, SA-β-Gal activity or lipofuscin deposits were not detected in the carotid artery of middle-aged mice. Previous studies reported that SMCs from aged human vessels or advanced atherosclerotic plaques express senescence markers in culture and exhibit prolonged population doubling times [35,36].

Although senescent cells are incapable of dividing, they continue to be metabolically active. In fact, senescent cells have been shown to release increased amounts of proinflammatory mediators, collectively known as SASP [37], which may affect neighboring cells. For example, cell lysates from old SMCs were found to stimulate target cell proliferation to a greater extent than lysates from young SMCs, and this effect could be blocked by antibodies against PDGF [38]. Aged SMCs isolated from non-human primates secreted significantly increased amounts of IL1β, MCP1, and TNFα compared to “young” control cells [39]. The enhanced SMC proliferation and migration and more pronounced neointima formation observed clinically and experimentally with age, also in our study, could, therefore, be the result of SMC senescence and paracrine stimulation of non-senescent cells.

Signals from the outer layer of the vessel wall also may affect SMC behavior. Neointima formation involves the migration of (myo)fibroblasts from the adventitia towards the neointima [11] and removal of the adventitia increased neointima formation [40]. The latter could be prevented by transplantation of subcutaneous adipose tissue from lean, but not from diet-induced obese mice or transplantation of visceral fat [16], suggesting that perivascular adipose tissue exerts protective effects that are lost upon exposure to risk factors. We and others have shown that diet-induced obesity enhances adventitial inflammation and that transplantation of visceral fat from obese mice promotes luminal stenosis [13,14,16]. Increased expression of leptin, MCP1, or TNFα and reduced levels of adiponectin could be identified as potential mediators underlying the neointima-promoting effects of obese perivascular fat [14,23,41]. Of note, visceral or subcutaneous fat was transplanted in these previous studies, which differs from local perivascular adipose tissue in several aspects [42]. In the present study, transplantation of PVAT obtained from the thoracic aorta of middle-aged donor mice did not promote neointima formation and also did not phenocopy the effects of age on this process. Of note, immunoincompetent nu/nu mice lacking mature CD4+ cells were used in the transplantation experiments, and differences in perivascular T cell accumulation compared to wild-type mice may have affected the vascular response to injury [43]. Regarding specific paracrine mediators, TNFα increased only in VAT but not in PVAT obtained from middle-aged mice, and aging did not alter the expression of MCP1, neither in VAT nor PVAT, whereas the perivascular expression of presumably protective mediators, such as adiponectin [16], increased with age. Although the absence of age-dependent effects of PVAT transplantation on neointima formation suggests that these paracrine mediators are not important, global changes in expression patterns and the interplay of mediators may be more relevant than alterations of a single factor. The age-dependent increase of other proinflammatory mediators secreted from senescent cells, such as IL1α, MIP1α, and MMP10, was also absent or blunted in thoracic PVAT compared to VAT isolated from the same mice, suggesting that PVAT (at least that surrounding the thoracic aorta) is protected from age-associated detrimental changes in gene expression patterns. A previous study reported an increased expression of inflammatory markers, including TNFα and MIP1α, in PVAT of middle-aged mice; however, only in those with diet-induced obesity [44].

Whereas a number of studies examined the impact of obesity on perivascular adipose tissue, only a few have looked into the effects of age on the morphology and paracrine activities of the adventitia, including perivascular adipose tissue. For example, aging and diet-induced obesity enhanced the ability of periaortic fat from WKY rats to stimulate human SMC proliferation in a paracrine manner [45]. Inflammatory factors secreted from PVAT of obese aged mice promoted oxidative and proinflammatory phenotypic alterations in the vascular wall of young control mice [44,45]. We now extend those previous findings by showing the functional consequences in terms of neointima formation. We found that transplantation of PVAT harvested from the murine thoracic aorta of both adult and middle-aged mice reduced neointima area and lumen stenosis in host mice, suggesting that thoracic PVAT exerts age-independent protective effects.

Cytokine stimulation induces tyrosine phosphorylation of STAT1, a transcription factor involved in inflammation, but also adipocyte differentiation [28]. Overactivation of STAT1 is counterregulated by PIAS1, which interacts with STAT1 dimers to block DNA binding and STAT1-mediated gene activation [25]. PIAS1 also negatively regulates NFkB signaling [26], and thus represents a key nodal negative regulator of inflammatory signaling. The observed higher expression of PIAS1 in PVAT surrounding the thoracic aorta of adult and aged may explain the observed blunted expression of proinflammatory markers in this fat depot and age-independent reduction in neointima formation, whereas PIAS1 expression was reduced in PVAT of patients with atherosclerotic vessel disease. A previous study has shown that PIAS1 is downregulated in white adipose tissue of mice with prediabetes, whereas overexpression of PIAS improved insulin sensitivity and visceral adipose tissue inflammation [46]. With regard to vascular disease, PIAS1-mediated sumoylation of RunX2 or ERK5 and p53 has been implicated in the reversal of vascular calcification [47] or endothelial dysfunction [48,49], respectively. Of note, global PIAS1 deficiency in mice is associated with increased perinatal mortality and reduced body weights [50,51], and the effects of perivascular transplantation of adipose tissues from those animals could not be examined.

## 4. Materials and Methods

### 4.1. Animals

C57BL/6JRj wild-type (WT) mice were from JANVIER LABS and housed at a constant temperature of 21 °C at a 12-h light/dark cycle. Mice were fed normal rodent chow (NC; 9% of energy from fat; Ssniff Spezialdiäten GmbH) for 8 weeks until the age of 16 weeks (“adult”) or 52 weeks (“middle-aged”). In one set of mice (*n* = 20 adult and *n* = 20 middle-aged mice), whole blood, adipose and vascular tissues were harvested for histological or protein and RNA analysis, respectively. In a second set of mice (*n* = 9 adult and *n* = 9 middle-aged mice), vascular injury was induced. Immunodeficient NMRI nu/nu mice (NMRI-Foxn1nu/Foxn1nu; The Jackson Laboratory) were used as recipients in PVAT transplantation experiments. Only male mice were examined throughout the entire study to avoid estrogen cycle dependent effects. All animal experiments had been approved by the institutional Animal Research Committee (Landesuntersuchungsamt Rheinland-Pfalz, 23 177-07/G13-081-1; 16/10/2013), and all animal procedures conformed to the guidelines from Directive 2010/63/EU (22 September 2010) of the European Parliament on the protection of animals used for scientific purposes. Euthanasia was performed in anesthetized mice by cervical dislocation.

### 4.2. Serum Analysis

Whole blood was collected by cardiac puncture from mice after an overnight fast (from 5 p.m. to 8 a.m. with drinking water ad libidum). Mice were anesthetized by intraperitoneal injection of 2% xylazine (Bayer; 6 mg/kg body weight) and 10% ketamine hydrochloride (Hameln Pharma plus; 100 mg/kg body weight). Serum was obtained by centrifugation (3000 rpm for 10 min) and stored at −80 °C. Fasting glucose and cholesterol (LDL, HDL, and total) levels were measured using colorimetric assays (BioAssay Systems, Hayward, CA, USA), fasting insulin (Crystal Chem, Zaandam, Netherlands) and leptin (R&D Systems Inc., Abingdon, UK) levels using enzyme-linked immunoassays. The HOMA-IR (homeostasis model assessment of insulin resistance) index was calculated using the formula: fasting insulin (µIU/mL) * fasting glucose (mg/dL)/405.

### 4.3. Vascular Tissue Harvest and Characterization

Vascular tissues (carotid artery, aorta) were harvested from anesthetized mice and either fixed in 4% zinc formalin (Sigma-Aldrich,St. Louis, MO, USA) and embedded in paraffin (Surgipath^®^ Paraplast; Leica Biosystems Inc., Buffalo Grove, IL, USA) or in 4% paraformaldehyde (Sigma-Aldrich) followed by 30% and 15% sucrose and embedded in Tissue-Tek^®^ O.C.T. compound (Sakura via Science Services GmbH, Munich, Germany). Sections were stained with hematoxyline and eosin (H&E) to visualize cell nuclei (blue-black), with Verhoeff’s Elastica stain (VES) to visualize elastic fibers (black) or with a combined Masson Trichrome (MTC)–VES stain to visualize muscle fibers (red) and extracellular matrix (blue). Senescent cells were visualized using a kit to detect SA-β-Gal activity (Abcam) or sudan black B (Sigma; 0.1% in 70% ethanol) to detect the accumulation of lipofuscins, according to [52].

### 4.4. Adipose Tissue Harvest and Characterization

Visceral (perigonadal) adipose tissue (VAT), brown (interscapular) adipose tissue (BAT) and PVAT surrounding the thoracic aorta were excised from anesthetized mice. Of each sample, one half was fixed in 4% zinc formalin and embedded in paraffin. Five µm-thick serial cross sections were stained with H&E and the single adipocyte area was determined using an Olympus BX51 microscope and image analysis software (Image-Pro Plus, version 7.0; Media Cybernetics Inc., Abingdon. UK). At least 10 adipocytes per microscope field (*n* = 3 per fat depot) and mouse (*n* = 3 per age group) were evaluated and the results averaged. The other half of the sample was transferred to TRI Reagent^®^ Solution (Invitrogen, Waltham, MA, USA) and stored at −80 °C pending RNA or protein isolation.

### 4.5. Carotid Artery INJURY

Mice were anesthetized by intraperitoneal injection of 2% xylazine/10% ketamine hydrochloride. Metamizole (ratiopharm, Ulm, Germany) was added to the drinking water (at a concentration of 2 mg/mL) for three days as analgetic. Carotid artery injury and neointima formation were induced using 10% ferric chloride (FeCl_3_) [14,53].

### 4.6. Perivascular Adipose Tissue Harvest and Transplantation

To obtain vital adipose tissue for subsequent perivascular transplantation, WT adult or middle-aged donor mice were anesthetized by intraperitoneal injection of 2% xylazine/10% ketamine hydrochloride. The PVAT surrounding the thoracic aorta was carefully removed, transferred into 0.9% sterile saline solution, and placed on ice. Immediately following vascular injury, PVAT freshly harvested from one donor was placed around the injured carotid artery of an immunodeficient NMRI nu/nu host.

### 4.7. Morphometry of Neointima Formation

Three weeks after vascular injury, mice were anesthetized and killed by perfusion with 4% zinc formalin fixative over the left ventricle. The injured (left) and uninjured (right) carotid artery were harvested, postfixed in zinc formalin and embedded in paraffin. Serial cross sections were stained with VES. The area of the neointima, media, adventitia and total vessel, and the degree of luminal stenosis were determined using Image-Pro Plus. The degree of luminal stenosis was calculated using the formula 100-A/B * 100, where A represents the area of the free vascular lumen and B the area encircled by the internal elastic lamina. At least three sections equally spaced through the injured arterial segment were evaluated and the results averaged per mouse.

### 4.8. Immunohistochemistry

Following deparaffinization through a series of graded alcohols, endogenous peroxidase activity was quenched in 3% H_2_O_2_ (in methanol; Carl Roth GmbH + Co. KG, Karlsruhe, Germany). Unspecific antigen-binding sites were blocked using 10% normal serum (in PBS; Abcam, Cambridge, UK), endogenous biotin was inhibited using the Avidin/Biotin Blocking kit (Vector Laboratories, Burlingame, CA, USA) followed by heat-induced epitope retrieval (0.01 M citrate buffer, pH 6.0; 800 W for 6 min). Sections were incubated overnight at 4 °C with antibodies directed against proliferating cell nuclear antigen (PCNA; Abcam), protein inhibitor of activated STAT1 (PIAS1; Abcam) or smooth muscle α-actin (SMA; Sigma). The next day, sections were incubated with secondary antibody (Molecular Probes Inc., Eugene, OR, USA), preformed avidin–biotin complexes (Vector Laboratories) and the peroxidase substrate 3-amino-9-ethylcarbazole (for PIAS and SMA) or 3, 3 -diaminobenzidine (for PCNA) (both Vector Laboratories) until color development. Sections were briefly counterstained with Gill’s hematoxyline (Sigma), mounted in ImmuMount (ThermoScientific, Waltham, MA, USA) and photographed (Olympus BX51 microscope, Olympus Europa SE & Co. KG, Hamburg, Germany).

### 4.9. Isolation and Analysis of Primary Smooth Muscle Cells

Primary murine SMCs were isolated from the thoracic aorta of adult and middle-aged mice, as described [15], and cultivated in DMEM/F12 Glutamax medium (Gibco by Life Technologies, Waltham, MA, USA) containing 10% fetal bovine serum (FBS, Gibco), 100 U/mL penicillin, 100 μg/mL streptomycin and 100 μM ascorbic acid. Experiments were performed in cells between passage 3 and 7. To examine SMC migration, the scratch-wound assay was employed [53].

### 4.10. Adipogenic Differentiation and Analysis of Murine 3T3L1 Fibroblasts

Murine 3T3-L1 fibroblasts (ATCC^®^, Manassas, VA, USA) were cultured in Dulbecco’s modified Eagle’s medium (DMEM; Gibco) supplemented with 10% FBS (Life Technologies, Waltham, MA, USA) and penicillin/streptomycin (Gibco). Cells were grown to confluency and differentiated into adipocytes using adipogenic medium supplemented with 0.5 mM methyl-isobutyl-xanthine (Sigma-Aldrich, company, city, country), 1 µM dexamethasone (Sigma-Aldrich), 10 µM insulin (Sigma-Aldrich), and 100 µM indomethacin (Sigma-Aldrich). After cultivation for 3 days, cells were switched to DMEM containing 10 μM insulin for 1 day (one cycle). Preadipocytes were transfected with 60 pmol of mouse PIAS1 siRNA (sc-36220) or control siRNA, either as fluorescein conjugate (sc-36869) or unconjugated (sc-37007; all Santa Cruz Biotechnology, Santa Cruz, CA, USA) using lipofectamine^®^ RNAiMAX transfection reagent (Thermo Fisher Scientific; Waltham, MA, USA) and analyzed 48 h later. Cells were fixed in 10% formaldehyde solution (Roth), stained with 0.5% Oil red O (Sigma) and examined under an inverted light microscope (Motic AE31, Motic, Wetzlar, Germany).

### 4.11. RNA Isolation and PCR Analysis

Total RNA from murine adipose tissue was isolated using TRI Reagent^®^ solution. The concentration and quality of the isolated RNA was checked by spectrophotometry before being reverse transcribed into cDNA using M-MLV reverse transcriptase (Promega Corporation, Madison, WI, USA). Quantitative real-time RT-PCR (qRT-PCR) was performed using SYBR^®^ Green (BioRad Laboratories, Hercules, CA, USA) and the real-time PCR StepOnePlus™ (Applied Biosystems, Foster City, CA, USA) system. Results were quantified using the ΔΔCt method with the reference genes β-actin (ACTA; for aortic tissue) or ribosomal protein lateral stalk subunit P0 (RPLP0; for adipose tissue) for normalization. Primer sequences are listed in Supplemental Table S2.

### 4.12. Protein Isolation and Western Blot Analysis

Murine adipose tissue homogenates were lysed in RIPA buffer (Cell Signaling Technology, Danvers, MA, USA) containing 1 mM phenylmethanesulfonyl fluoride (Cell Signaling Technology). Equal amounts of protein were fractionated by SDS polyacrylamide gel electrophoresis and transferred to nitrocellulose membranes (Whatman GmbH, Dassel, Germany). Membranes were blocked in 5% BSA (in TBS/0.1% Tween-20) followed by incubation with antibodies against leptin (abcam), myeloid differentiation primary response protein-88 (MyD88; abcam), p53 (Santa Cruz Biotechnology), perilipin (PLIN; Cell Signaling Technology), PIAS1 (Cell Signaling Technology), preadipocyte factor-1 (PREF1; Thermo Fisher Scientific), or suppressor of cytokine signaling-1 (SOCS1; Novus Biologicals, Centennial, CO, USA). Antibodies against glyceraldehyde-3-phosphate dehydrogenase (GAPDH; Hy Test Ltd., Turku, Finland) were used to show total protein loading. Protein bands were visualized using horseradish peroxidase-conjugated secondary antibodies (Amersham Biosciences, Chalfont St. Giles, UK) and detected with enhanced chemiluminescence substrate (Pierce Manufacturing, Appleton, WI, USA).

### 4.13. Human (Perivascular) Adipose Tissue Samples

Sixteen patients (100% male, mean age, 69.8 ± 2.2 years) undergoing surgical repair of an abdominal aortic aneurysm (AAA), 10 patients (80% male, mean age, 67.0 ± 2.3 years) with coronary artery disease (CAD) undergoing coronary artery bypass graft surgery, and 9 patients (56% male, mean age, 66.7 ± 10.9 years) with aortic valve stenosis undergoing surgical replacement were included in the study. The study complied with the declaration of Helsinki and was approved by the Ethics Committee of the University General Hospital of Alexandroupolis, Greece. All patients provided written informed consent prior to study inclusion. PVAT was collected perioperatively and immediately transferred to ice-cold saline solution.

### 4.14. Enzyme-Linked Immunosorbent Assay

The amount of PIAS1 protein expressed in human adipose tissue protein lysates was quantified using an ELISA kit (Elabscience Biotechnology Inc., Houston, TX, USA), following the manufacturer’s instructions. Tissues were lysed in 1X RIPA buffer (Cell Signaling Technology) containing 1X protease and phosphatase inhibitor cocktail (Thermo Fisher Scientific). A standard amount of total protein (100 μg in 100 μL) was loaded into each well. Results are expressed per mg total adipose tissue protein.

### 4.15. Statistical Analysis

Quantitative data are reported as mean ± standard deviation (SD). Normal distribution was examined using the D’Agostino & Pearson omnibus normality test. Two groups were compared using Student’s *t*-test if normal distribution was present. If not or if sample size was below *n* = 5, a nonparametric Mann–Whitney test was used. If more than two groups were compared, the one-way analysis of variance (ANOVA) test followed by Sidak’s multiple comparison test or the Kruskall–Wallis test followed by Dunn’s multiple comparison testing were performed. Paired analysis was employed to compare findings in different adipose tissues from the same patient. All analyses were performed using GraphPad PRISM data analysis software (version 7.0 for Windows; GraphPad Software Inc., San Diego, CA, USA).

## 5. Conclusions

In conclusion, our findings suggest that age-associated systemic and vessel wall alterations are key determinants of the vascular response to injury and that age affects adipose tissue and its paracrine activities on the vessel wall in a depot-specific manner. They also support a critical role of PIAS1 in mediating the age-independent vasculoprotective effects of PVAT and suggest that reduced perivascular expression of PIAS1 may play a role in the development of human atherosclerotic vessel disease.

## Figures and Tables

**Table 1:** Body and adipose tissue weight and fasting serum parameters in adult and middle-aged C57BL/6J wild-type mice.

Parameter	Adult	Middle-Aged
number	20	20
sex	male	male
age (weeks)	16	52
diet	10% NC	10% NC
body weight (g)	32.0 ± 0.98	36.7 ± 0.83 ***
BAT weight (mg)	0.34 ± 0.04	0.30 ± 0.03
PVAT weight (mg)	n.d.	n.d.
SCATax weight (mg)	0.29 ± 0.04	0.35 ± 0.05
SCATing weight (mg)	0.52 ± 0.06	0.65 ± 0.08
VAT weight (mg)	0.74 ± 0.10	1.12 ± 0.14 *
number	15	15
leptin (ng/mL)	12.1 ± 3.9	13.1 ± 4.0
glucose (mg/dL)	160 ± 19	232 ± 21 *
insulin (µIU/mL)	6.2 ± 0.7	5.1 ± 0.2
HOMA-IR	2.6 ± 0.5	2.9 ± 0.3
total cholesterol (mg/dL)	103 ± 4.7	106 ± 6.7
HDL cholesterol (mg/dL)	82 ± 4.7	80 ± 4.9
LDL cholesterol (mg/dL)	17 ± 2.0	15 ± 1.2

Data are given as mean ± SEM. * *p* < 0.05 and *** *p* < 0.001 vs. adult mice, as determined by Student’s *t*-test. Abbreviations: ax, axillar; BAT, brown adipose tissue; HDL, high-density lipoprotein; HOMA-IR, homeostasis model assessment of insulin resistance; ing, inguinal; LDL, low-density lipoprotein; NC, normal chow; n.d., not determined; SCAT, subcutaneous adipose tissue; VAT, visceral adipose tissue.

## Supplementary Materials

Supplementary materials can be found at https://www.mdpi.com/1422-0067/21/1/282/s1.

## Abbreviations

AAA	abdominal aortic aneurysm
AS	aortic valve stenosis
AT	adipose tissue
BAT	brown adipose tissue
CAD	coronary artery disease
FBS	fetal bovine serum
HOMA-IR	homeostasis model assessment of insulin resistance
MTC	Masson trichrome
NC	normal chow
PBS	phosphate-buffered saline
PVAT	perivascular adipose tissue
SASP	senescence-associated secretory phenotype
SMC	smooth muscle cell
Tx	transplantation
VAT	visceral adipose tissue
VES	Verhoeff’s elastic stain
WT	wild-type
