# Supplementary material for: Age-Dependent and -Independent Effects of Perivascular Adipose Tissue and Its Paracrine Activities during Neointima Formation
Source: Int J Mol Sci. 2019 Dec 31;21(1):282. doi: 10.3390/ijms21010282 (PMC6981748; doi:10.3390/ijms21010282)
Supplement: Supplementary file 1 [file ijms-21-00282-s001.pdf]

# SUPPLEMENTAL MATERIAL

## Supplemental Figures and Figure Legends

### Supplemental Figure S1

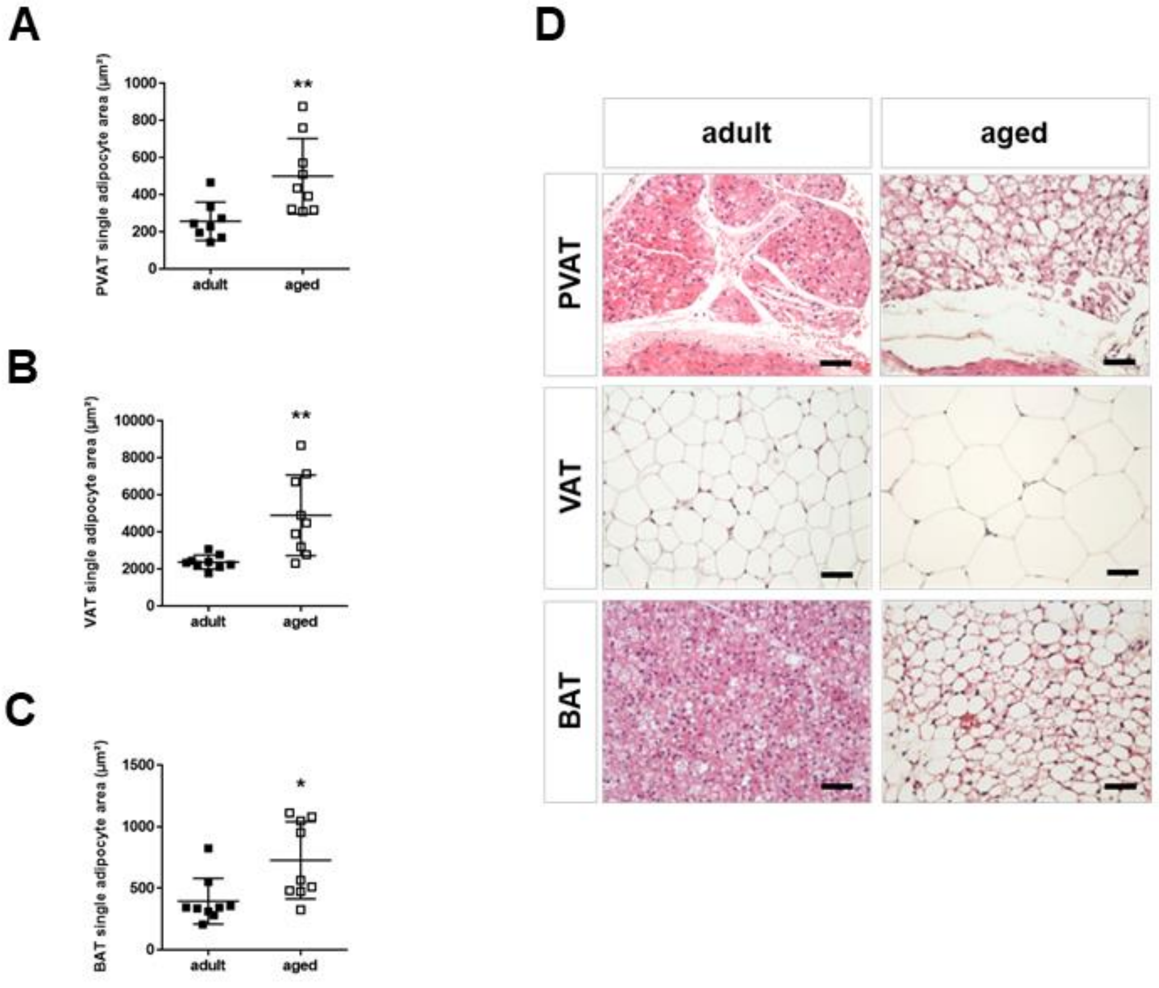


**Supplemental Figure S1. Effect of age on murine adipose tissue depots.** Perivascular (PVAT), visceral (VAT) and brown (BAT) adipose tissue was harvested from adult (n=9) and aged (n=9) C57BL/6Jrj mice, paraffin-embedded and 5 µm-thick cross-sections prepared for histological analysis. The mean single adipocyte area was determined on H&E-stained cross sections. The results of the quantitative analysis for PVAT are shown in (**A**), for VAT in (**B**) and for BAT in (**C**). Individual data points and the mean ± SD are shown. *P<0.05 and **P<0.01 vs. adult mice, as determined using Student's t test for unpaired means in panels A and B or Mann-Whitney test in panels C. Representative findings are shown in (**D**). Size bars represent 100 µm.

## Supplemental Figure S2

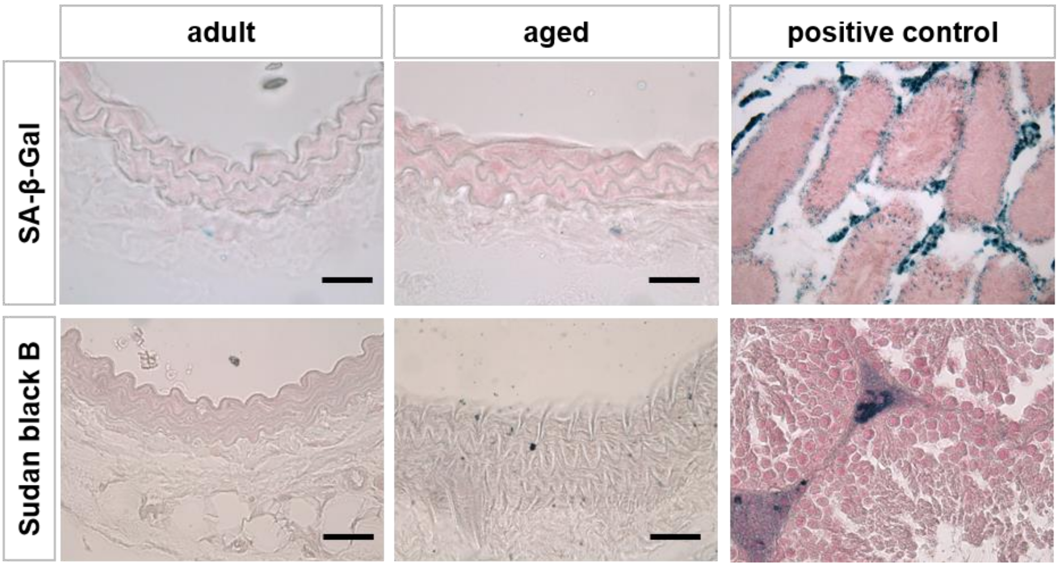


**Supplemental Figure S2. Histochemical detection of senescent cells.** Repressentative cross-sections through carotid arteries of adult (16 weeks-of-age) and aged (52 weeks-of-age) mice after histochemical analysis of the presence of senescence-associated-β-galactosidase (SA-β-Gal) activity or lipofuscin (using Sudan black B stain) to visualize senescent cells are shown. Size bars indicate 25 µm. Results of staining of sections through testis of aged mice (positive control) are also shown.

## Supplemental Figure S3

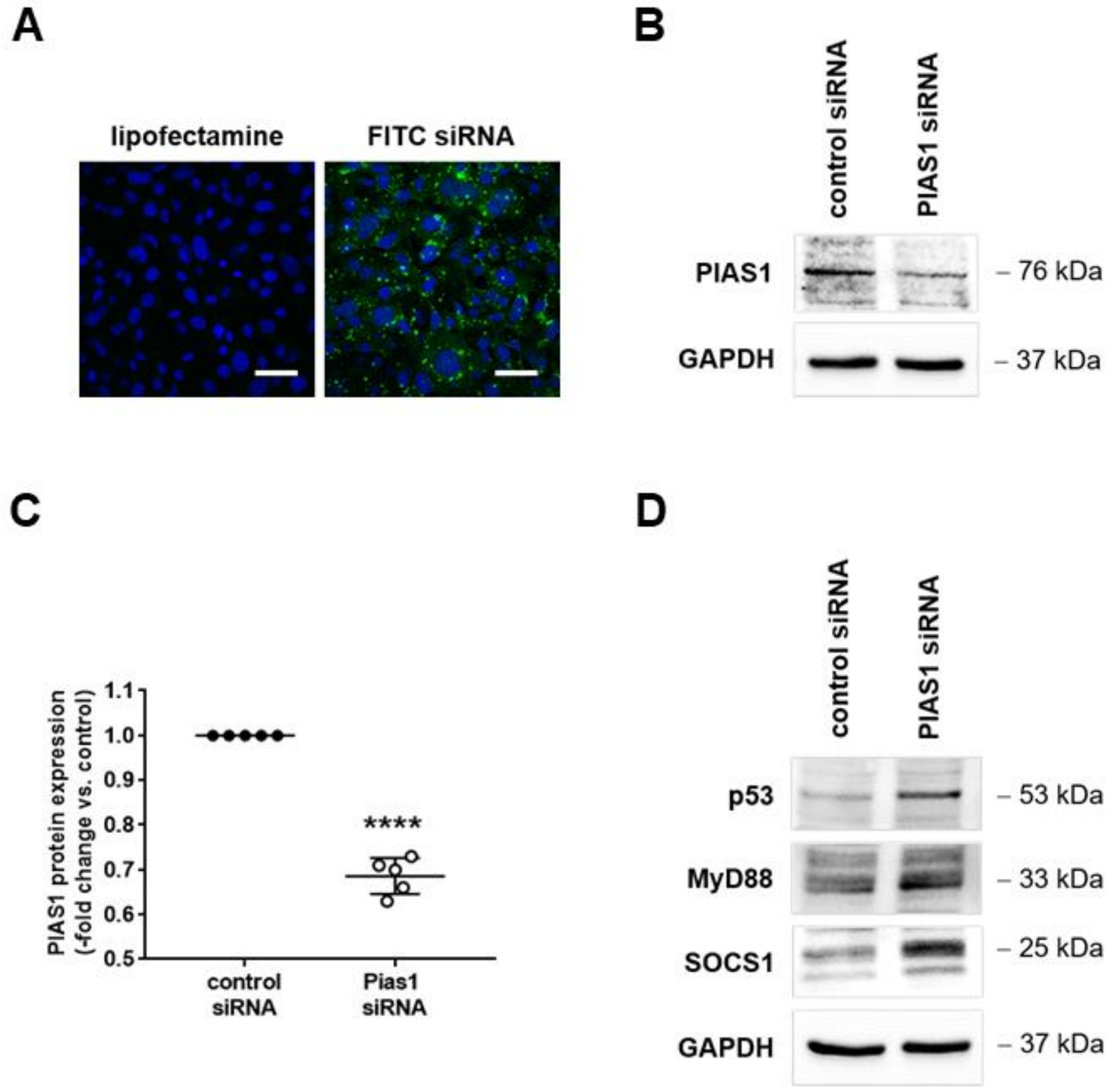


**Supplemental Figure S3. Transfection of murine preadipocytes with siRNA.** Murine 3T3L1 preadipocytes were cultivated and transfected with FITC-labeled control siRNA (FITC siRNA) or treated with lipofectamine alone, as described in the Methods, and the cellular transfection efficacy examined 48 hours later under the confocal microscope. Size bars represent 10 µm. (**A**). 3T3L1 preadipocytes were transfected with unlabeled control siRNA or siRNA targeting murine PIAS1 and the effects on the expression of PIAS1 (**B**, quantification in **C**) or its downstream targets p53, MyD88 and SOCS1 examined (**D**).

## Supplemental Figure S4

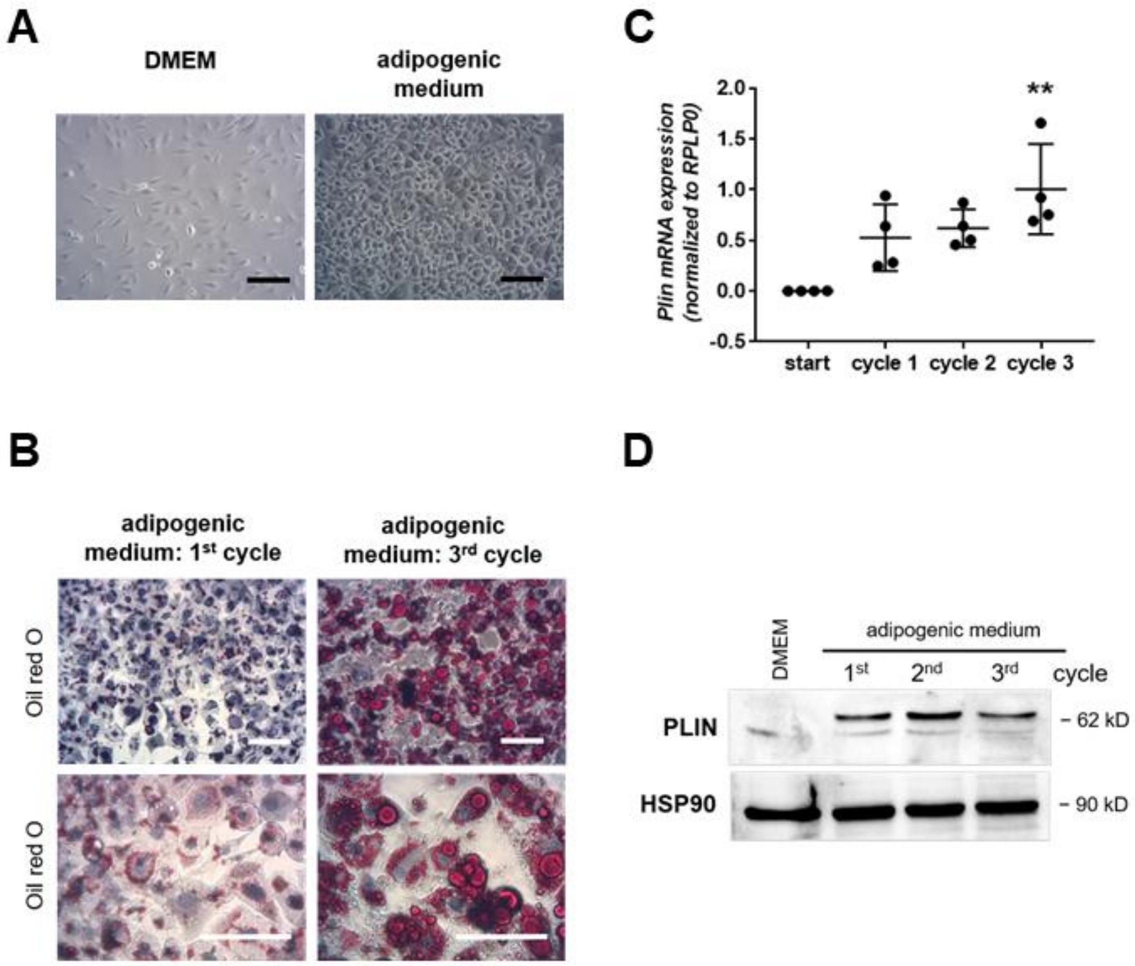


**Supplemental Figure S4. Murine adipocyte differentiation *in vitro*.** Murine 3T3L1 preadipocytes were differentiated over three cycles into adipocytes, as described in the Methods, and analyzed under the inverted microscope, unstained (**A**) and following Oil red O staining of lipids (**B**). Size bars represent 10 µm. Total RNA or protein were isolated and analyzed for the expression of perilipin (Plin) mRNA (**C**) and protein (**D**) using *real time* PCR (n=4 independent experiments) or western blot (a representative membrane from n=6 independent experiments is shown), respectively. **P<0.01 vs. cells at start of the experiment (as determined by Kruskall-Wallis followed by Dunn's multiple comparisons test). Individual data points and the mean ± SD are shown.

## Supplemental Figure S5

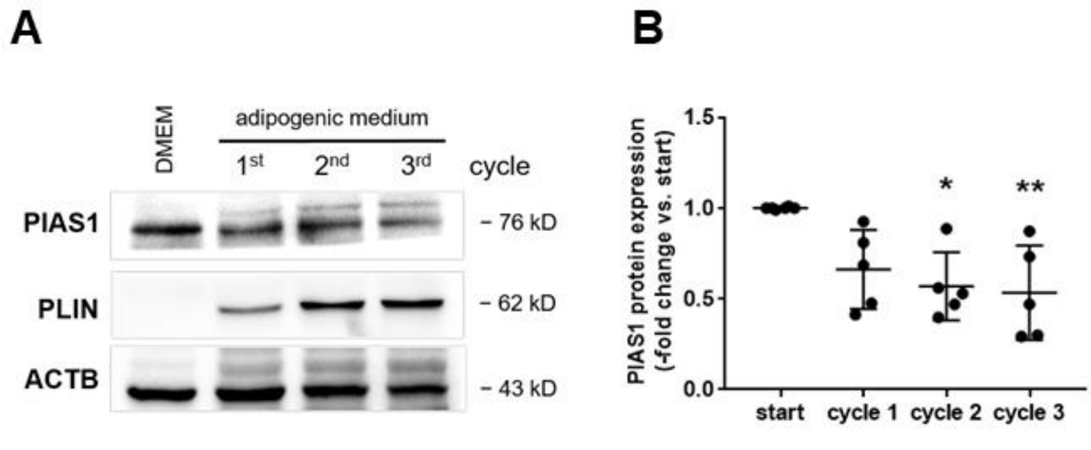


**Supplemental Figure S5. Expression of PIAS1 in murine preadipocytes.** Murine 3T3L1 fibroblasts were differentiated into adipocytes, as described in the Methods, and changes in the expression of PIAS1 and perilipin (Plin) examined (n=4 independent experiments). A representative western blot membrane is shown in (**A**), the results of the quantitative analysis of PIAS1 protein levels in (**B**). Individual data points and the mean ± SD are shown. *P<0.05 and **P<0.01 vs. start (as determined by Kruskall-Wallis followed by Dunn’s multiple comparisons test).

## Supplemental Figure S6

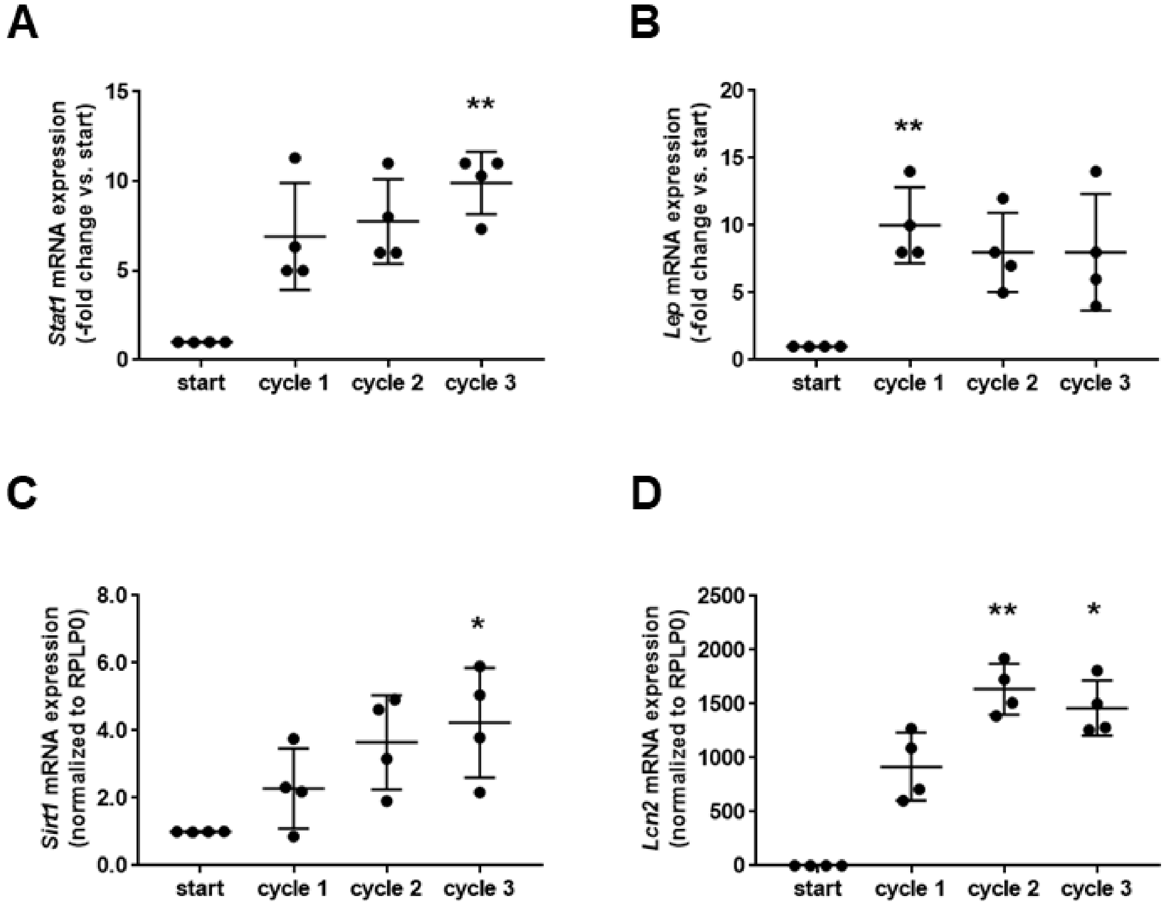


**Supplemental Figure S6. Expression of STAT1 and STAT1-regulated genes during murine adipocyte differentiation *in vitro*.** Murine 3T3L1 preadipocytes were differentiated over three cycles into adipocytes and *real-time* PCR analysis was performed to determine changes in mRNA expression levels of STAT1 (**A**) and STAT1 target genes involved in adipocyte differentiation, such as leptin (*Lep*; **B**), sirtuin-1 (*Sirt1*; **C**) and lipocalin-2 (*Lcn*2; **D**). Individual data points and the mean ± SD are shown. *P<0.05 and **P<0.01 vs. cells at the start of the experiment (as determined by Kruskall-Wallis followed by Dunn's multiple comparisons test; n=4 independent experiments).

## Supplemental Figure S7

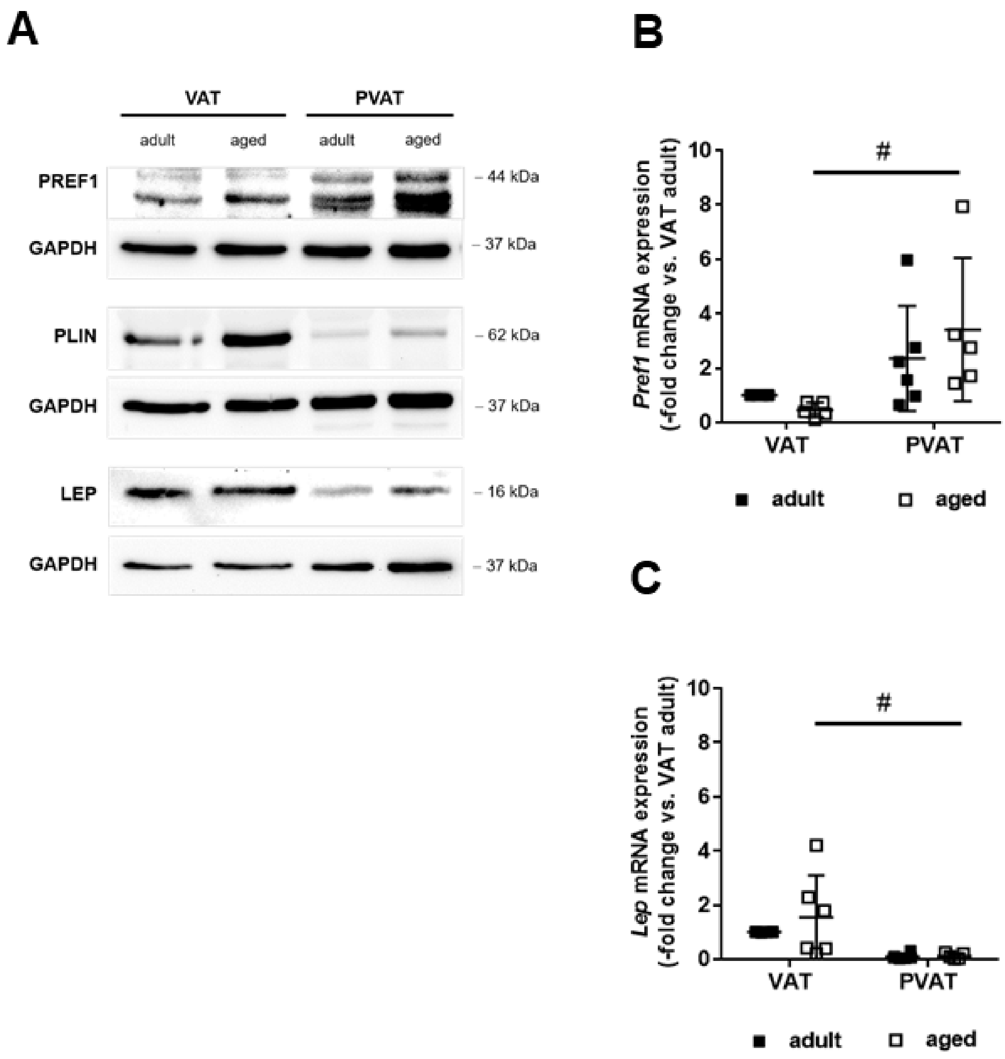


**Supplemental Figure S7. Expression of adipocyte differentiation markers in visceral and perivascular adipose tissue of adult and middle-aged mice.** Perivascular adipose tissue (PVAT) was harvested from adult and middle-aged mice and the protein expression of the preadipocyte marker PREF1, the mature adipocyte marker perilipin (PLIN) and the adipokine leptin (LEP) examined. A representative western blot membrane is shown in (**C**). Pref1 (**D**) and leptin (**E**) mRNA expression was examined using real-time PCR (n=5-6 mice per group). Individual data points and the mean ± SD are shown. #P<0.05 vs. VAT of mice of the same age group (as determined by Kruskall-Wallis followed by Dunn’s multiple comparisons test).

## Supplemental Tables

**Supplemental Table S1. Mice used for perivascular adipose tissue transplantation**

| | control | PVAT-Tx adult | PVAT-Tx aged |
|---|---|---|---|
| **host mice** | **NMRI nu/nu** | **NMRI nu/nu** | **NMRI nu/nu** |
| **number** | 11 | 9 | 8 |
| **sex** | male | male | male |
| **age (weeks)** | 10 | 10 | 10 |
| **body weight (g)** | 35.7±0.6 | 36.0±0.6 | 35.2±0.5 |
| **diet** | NC | NC | NC |
| **donor mice** | | **C57BL/6J** | **C57BL/6J** |
| **number** | - | 9 | 8 |
| **sex** | - | male | male |
| **age (weeks)** | - | 13 | 52 |
| **body weight (g)** | - | 24.8±0.4 | 31.9±0.7 *** |
| **diet** | - | NC | NC |

Data are given as mean±standard error of the mean (SEM). ***P<0.001 vs. NMRI nu/nu PVAT-Tx adult mice, as determined by Student's t-test. *Abbreviations:* NC, normal chow; PVAT, perivascular adipose tissue; Tx, transplantation.

**Supplemental Table S2. Murine primer sequences**

| Gene | primer sequence (in 5‘ – 3‘ direction) | product length (bp) | reference |
|---|---|---|---|
| ***Ace2*** | F: TCTGGGCAAACTCTATGCTGACT<br>R: GGCTGTCAAGAAGTTGTCCATTG | 72 | 1 |
| ***Actb*** | F: GCAGGAGTACGATGAGTCCG<br>R: ACGCAGCTCAGTAACAGTCC | 74 | NM_007393.5 |
| ***Adipoq*** | F: GATGGCAGAGATGGCACTCC<br>R: CTTGCCAGTGCTGCCGTCAT | 282 | 2 |
| ***Casp1*** | F: TGGTCTTGTGACTTGGAGGA<br>R: GGTCACCCTATCAGCAGTGG | 96 | NM_009807.2 |
| ***Ccl2*** | F: GGCTGGAGAGCTACAAGAGG<br>R: TCTTGAGCTTGGTGACAAAAAC | 75 | 3 |
| ***Ccnd1*** | F: GTTCGTGGCCTCTAAGATGAAGGA<br>R: CACTTGAGCTTGTTCACCAGAAGC | 129 | NM_007631.2 |
| ***Il1α*** | F: GCCTTATTTCGGGAGTCTAT<br>R: TAGGGTTTGCTCTTCTCTTACA | 158 | 5 |
| ***Il10*** | F: ATTTGAATTCCCTGGGTGAGAAG<br>R: CACAGGGGAGAAATCGATGACA | 75 | 6 |
| ***Il18*** | F: ACGTGTTCCAGGACACAACA<br>R: ACAAACCCTCCCCACCTAAC | 181 | 7 |
| ***Lcn2*** | F: TGCAAGTGGCCACCACGGAC<br>R: GCATTGGTCGGTGGGGACAGAGA | 203 | 8 |
| ***Lep*** | F: GAGACCTCCTCCATGTGCTG<br>R: CATTCAGGGCTAAGGTCCAA | 191 | 9 |
| ***Mip1α*** | F: TGAGAGTCTTGGAGGCAGCGA<br>R: TGTGGGTACTTGGCAGCAAACA | 135 | 10 |
| ***Mmp3*** | F: GCATCCCCTGATGTCCTCGTGG<br>R: TCCCCGGAGGGTGCTGACTG | 111 | 11 |
| ***Mmp10*** | F: CCTGTGTTGTCTGTCTCTCCAAGA<br>R: CGTGCTGACTGAATCAAAGGAC | 78 | 12 |
| ***Mmp12*** | F: CCTCGATGTGGAGTGCCCGA<br>R: CTCACGCTTCATGTCCGGAGTG | 118 | 11 |
| ***p16*** | F: AAGAGCAGAGCTAAATCC<br>R: TTTCTCATGCCATTCCTT | 199 | 13 |
| ***p21*** | F: CTCCACAGATTTCTATCACTCCA<br>R: CTCCTGATATACGCTGCCTGC | 192 | AH011321.2 |
| ***p53*** | F: CACAGCGTGGTGGTACCTTA<br>R: TCTTCTGTACGGCGGTCTCT | 218 | 14 |
| ***Pref1*** | F: CGTGATCAATGGTTCTCCCT<br>R: AGGGGTACAGCTGTTGGTTG | 148 | 15 |
| ***Rplp0*** | F: AGCTGAAGCAAAGGAAGAGTCGGA<br>R: ACTTGGTTGCTTTGGCGGGATTAG | 84 | 16 |
| ***Sirt1*** | F: GCTGACGACTTCGACGACG<br>R: TCGGTCAACAGGAGGTTGTCT | 101 | 8 |
| ***Stat1*** | F: CTGAATATTTCCCTCCTGGG<br>R: TCCCGTACAGATGTCCATGAT | 103 | 17 |

| | | | |
|---|---|---|---|
| ***Tgfβ*** | F: CAGTGGCTGAACCAAGGAGAC<br>R: ATCCCGTTGATTTCCACGTG | 101 | 18 |
| ***Tnfα*** | F: CTGTAGCCCACGTCGTAGC<br>R: TTGAGATCCATGCCGTTG | 97 | 3 |

*Abbreviations:* Ace2, angiotensin converting enzyme-2; Actb, β-actin; Adipoq, adiponectin; Casp1, caspase-1; Ccl2, chemokine ligand-2; Ccnd1, cyclin D1; Il, interleukin; Lcn2, lipocalin-2; Lep, leptin; Mip1α, macrophage inflammatory protein 1-alpha; Mmp, matrix-metalloproteinase; p16, cyclin-dependent kinase inhibitor 2A; p21, cyclin-dependent kinase inhibitor 1; p53, tumor suppressor p53; Pref1, Preadipocyte factor 1; Rplp0, ribosomal protein lateral stalk subunit P0; Sirt1, sirtuin-1; Stat1, Signal transducer and activator of transcription 1; Tgfβ, transforming growth factor-beta; Tnfα, tumor necrosis factor-alpha.

## Supplemental References


1. Liu J, Ji H, Zheng W, Wu X, Zhu JJ, Arnold AP, Sandberg K. Sex differences in renal angiotensin converting enzyme 2 (ACE2) activity are 17beta-oestradiol-dependent and sex chromosome-independent. *Biol Sex Differ.* **2010**, 1, 6.
2. Takaoka M, Nagata D, Kihara S, Shimomura I, Kimura Y, Tabata Y, Saito Y, Nagai R, Sata M. Periadventitial adipose tissue plays a critical role in vascular remodeling. *Circ Res.* **2009**, 105, 906–911.
3. Rocha VZ, Folco EJ, Sukhova G, Shimizu K, Gotsman I, Vernon AH, Libby P. Interferon-gamma, a Th1 cytokine, regulates fat inflammation: a role for adaptive immunity in obesity. *Circ Res.* **2008**, 103, 467–476.
4. Lin DW, Chang IC, Tseng A, Wu ML, Chen CH, Patenaude CA, Layne MD, Yet SF. Transforming growth factor beta up-regulates cysteine-rich protein 2 in vascular smooth muscle cells via activating transcription factor 2. *J Biol Chem.* **2008**, 283, 15003–15014.
5. Sieber MW, Claus RA, Witte OW, Frahm C. Attenuated inflammatory response in aged mice brains following stroke. *PLoS One.* **2011**, 6, e26288.
6. Yee CS, Yao Y, Xu Q, McCarthy B, Sun-Lin D, Tone M, Waldmann H, Chang CH. Enhanced production of IL-10 by dendritic cells deficient in CIITA. *J Immunol.* **2005**, 174, 1222–1229.
7. Bhat OM, Kumar PU, Giridharan NV, Kaul D, Kumar MJ, Dhawan V. Interleukin-18-induced atherosclerosis involves CD36 and NF-kappaB crosstalk in Apo E-/- mice. *J Cardiol.* **2015**, 66, 28–35.
8. Zhao Y, Ling F, Griffin TM, He T, Towner R, Ruan H, Sun XH. Up-regulation of the Sirtuin 1 (Sirt1) and peroxisome proliferator-activated receptor gamma coactivator-1alpha (PGC-1alpha) genes in white adipose tissue of Id1 protein-deficient mice: implications in the protection against diet and age-induced glucose intolerance. *J Biol Chem.* **2014**, 289, 29112–29122.
9. Hoggard N, Hunter L, Lea RG, Trayhurn P and Mercer JG. Ontogeny of the expression of leptin and its receptor in the murine fetus and placenta. *Br J Nutr.* **2000**, 83, 317–326.
10. Denaes T, Lodder J, Chobert MN, Ruiz I, Pawlotsky JM, Lotersztajn S, Teixeira-Clerc F. The Cannabinoid Receptor 2 Protects Against Alcoholic Liver Disease Via a Macrophage Autophagy-Dependent Pathway. *Sci Rep.* **2016**, 6, 28806.
11. Johnson JL, Dwivedi A, Somerville M, George SJ, Newby AC. Matrix metalloproteinase (MMP)-3 activates MMP-9 mediated vascular smooth muscle cell migration and neointima formation in mice. *Arterioscler Thromb Vasc Biol.* **2011**, 31, e35–e44.
12. Hasebe Y, Egawa K, Shibanuma M, Nose K. Induction of matrix metalloproteinase gene expression in an endothelial cell line by direct interaction with malignant cells. *Cancer Sci.* **2007**, 98, 58–67.
13. Bailey-Downs LC, Tucsek Z, Toth P, Sosnowska D, Gautam T, Sonntag WE, Csiszar A, Ungvari Z. Aging exacerbates obesity-induced oxidative stress and inflammation in perivascular adipose tissue in mice: a paracrine mechanism contributing to vascular redox dysregulation and inflammation. *J Gerontol A Biol Sci Med Sci.* **2013**, 68, 780–792.
14. Mauro C, Leow SC, Anso E, Rocha S, Thotakura AK, Tornatore L, Moretti M, De SE, Beg AA, Tergaonkar V, Chandel NS, Franzoso G. NF-kappaB controls energy homeostasis and metabolic adaptation by upregulating mitochondrial respiration. *Nat Cell Biol.* **2011**, 13, 1272–1279.
15. Jensen B, Farach-Carson MC, Kenaley E, Akanbi KA. High extracellular calcium attenuates adipogenesis in 3T3-L1 preadipocytes. *Exp Cell Res.* **2004**; 301: 280–292.

16. Manka D, Chatterjee TK, Stoll LL, Basford JE, Konaniah ES, Srinivasan R, Bogdanov VY, Tang Y, Blomkalns AL, Hui DY and Weintraub NL. Transplanted perivascular adipose tissue accelerates injury-induced neointimal hyperplasia: role of monocyte chemoattractant protein-1. *Arterioscler Thromb Vasc Biol.* **2014**, 34, 1723–1730.
17. Shaul ME, Bennett G, Strissel KJ, Greenberg AS, Obin MS. Dynamic, M2-Like Remodeling Phenotypes of CD11c+ Adipose Tissue Macrophages During High-Fat Diet-Induced Obesity in Mice. *Diabetes.* **2010**, 59, 1171–1181.
18. Okada H, Kikuta T, Kobayashi T, Inoue T, Kanno Y, Takigawa M, Sugaya T, Kopp JB, Suzuki H. Connective tissue growth factor expressed in tubular epithelium plays a pivotal role in renal fibrogenesis. *J Am Soc Nephrol.* **2005**, 16, 133–143.
